# Magic Mathematical Relationships for Nanoclusters

**DOI:** 10.1186/s11671-019-2939-5

**Published:** 2019-05-02

**Authors:** Forrest H. Kaatz, Adhemar Bultheel

**Affiliations:** 1 Mesalands Community College,, 911 South 10th Street, Tucumcari, 88401 NM USA; 2 Department Computer Sci., KU Leuven, Celestijnenlaan 200A, Heverlee, 3001 Belgium

**Keywords:** Nanoclusters, Topological indices, Coordination, Magic numbers, Dispersion

## Abstract

Size and surface properties such as catalysis, optical quantum dot photoluminescense, and surface plasmon resonances depend on the coordination and chemistry of metal and semiconducting nanoclusters. Such coordination-dependent properties are quantified herein via “magic formulas” for the number of shells, *n*, in the cluster. We investigate face-centered cubic, body-centered cubic, simple cubic clusters, hexagonal close-packed clusters, and the diamond cubic structure as a function of the number of cluster shells, *n*. In addition, we examine the Platonic solids in the form of multi-shell clusters, for a total of 19 cluster types. The number of bonds and atoms and coordination numbers exhibit magic number characteristics versus *n*, as the size of the clusters increases. Starting with only the spatial coordinates, we create an adjacency and distance matrix that facilitates the calculation of topological indices, including the Wiener, hyper-Wiener, reverse Wiener, and Szeged indices. Some known topological formulas for some Platonic solids when *n*=1 are computationally verified. These indices have magic formulas for many of the clusters. The simple cubic structure is the least complex of our clusters as measured by the topological complexity derived from the information content of the vertex-degree distribution. The dispersion, or relative percentage of surface atoms, is measured quantitatively with respect to size and shape dependence for some types of clusters with catalytic applications.

## Introduction

Magic numbers and formulas for nanoclusters have a long history dating to the prescient publication by van Hardeveld and Hartog in 1969 [1]. Their insights predated the nanoscience era. Since then, we have seen magic numbers appear in 2D polygons and 3D polyhedra [2], carbon fullerenes [3], and in a limited scope again in clusters [4]. Such diverse materials such as silicon [5], boron [6], and in fact over 1000 publications from the indexing service “Web of Science” refer to magic numbers in clusters. The study of the size and shape of nanoclusters is important to today’s society, since this determines not only the intrinsic physical and chemical properties, but also the relevance for optical, catalytic, electronic, and magnetic applications [7]. Our aim is to update the database of this knowledge with current relationships and data, now that we have entered the nano realm.

The occurrence of magic numbers in nanoclusters has to do primarily with the formation of shells of atoms upon a fundamental cell. When the number of atoms completes a full shell, we find a unique set of numbers, termed “magic,” that defines the shells of atoms. A cluster is represented by a graph with the atoms as vertices and the bonds as edges. It consists of nested shells like layers of an onion. We define the numbers of layers as *n* and discover the mathematical relationships of nearest neighbor coordination numbers, bonds, the total number of atoms, and some topological indices as a function of *n*. The original paper by van Hardeveld and Hartog [1] considered fcc, bcc, and hcp clusters. The reference by Teo and Sloane [2] considers polyhedra and Platonic solids but neglects the relationship of nearest neighbor coordination numbers. We add to this database by looking at simple cubic, diamond cubic, and the Platonic solids, in addition to the topological properties and dispersion of the clusters.

The transition in size from bulk to clusters of a few atoms is really about the relationship of the surface atoms as compared to bulk atoms. Properties such as catalytic chemistry, surface plasmon resonance, and optical quantum dot photoluminescence [8] are affected by the coordination and number of surface atoms. The dispersion or relative percentage of surface atoms is determined by the ratio of surface atoms to the total number of atoms, as has been considered previously [9]. Our analysis will determine the relative ranking of the dispersion in terms of cluster geometry.

Topological indices started with Wiener’s original paper regarding his index and the boiling points of paraffin [10]. It was not until some time later that Hosoya introduced a mathematical formalism to analyze topological indices [11]. We have previously introduced topological indices and nanoclusters [12]. At this writing, there exist many indices, some of which depend on the adjacency or distance matrix. We show here that in many of the cluster shapes, magic mathematical relationships exist for the four indices as a function of *n* and the number of shells.

## Methods

For each of the types of clusters we study, we create a computational algorithm which determines the atomic coordinates of the clusters. We then proceed to create an adjacency matrix and a distance matrix defined as follows. An adjacency matrix **A** is created where we define *i* and *j* as nearest neighbors and separate them from the rest by requiring that *r*_*ij*_<*r*_*c*_, where *r*_*c*_ is a threshold value, slightly above the nearest neighbor distance, but less than the second neighbor distance. Thus,

1 $$ \mathbf{A}(i,j)=\left\{\begin{array}{l} 1~~ \text{if}~ r_{ij}< r_{c} ~\text{and}~ i\ne j\\ 0~~ otherwise \end{array}\right.   $$

where *r*_*ij*_ is the Euclidean distance between atom *i* and atom *j*. An appropriate value for *r*_*c*_ is 1.32·*r*_*min*_, where *r*_*min*_ is the smallest bond length. This applies to the dodecahedral structure, as well as the others we study. The coordination numbers of the cluster are simply the number of non-zero elements in a column of the adjacency matrix. The distance matrix is defined as

2 $$ \mathbf{D}(i,j) = \left\{\begin{array}{ll} 0 & i = j \\ d_{ij} & i \neq j \end{array}\right.   $$

where *d*_*ij*_ is the length of the shortest path in the graph from *i* to *j*. An efficient algorithm for the calculation of the distance matrix from the adjacency matrix exists [13]. Using these definitions, we can calculate the Wiener index, *W*(*G*), the hyper-Wiener index, *WW*(*G*), the reverse Wiener index *rW*(*G*), and the Szeged index, *Sz*(*G*), as previously detailed [14]. These calculations use the the same algorithm that we have previously used for topological indices and nanoclusters [12].

Previous authors have offered proofs of magic relationships, which we condense in our notation, relevant for the work presented here [1, 2]. Since we create nearest neighbor adjacency matrices, we know the coordination number *cn*_*i*_ of vertex *i* by summing the elements of **A**(*i*,:). Our structure consists of *n*+1 shells numbered 0,1,…,*n*. Let $\phantom {\dot {i}\!}N_{{cn}_{i}}(n)$ be the number of atoms with coordination *cn*_*i*_ where 1≤*cn*_*i*_≤*cn*_*M*_ with *cn*_*M*_ the maximal coordination in the cluster. Then the total number of atoms in the cluster is given by

3 $$ N_{T}(n) = \sum_{{cn}_{i}=1}^{{cn}_{M}}{N_{{cn}_{i}}(n)}.   $$

The surface atoms in the outer shell *n* have a set of bondings less than the bulk coordination. Thus the maximal coordination for surface atoms is *cn*_*s*_<*cn*_*M*_, and the number of surface atoms is

4 $$ N_{S}(n) = \sum_{{cn}_{i}=1}^{{cn}_{s}}{N_{{cn}_{i}}(n)}.   $$

**Fig. 1:**
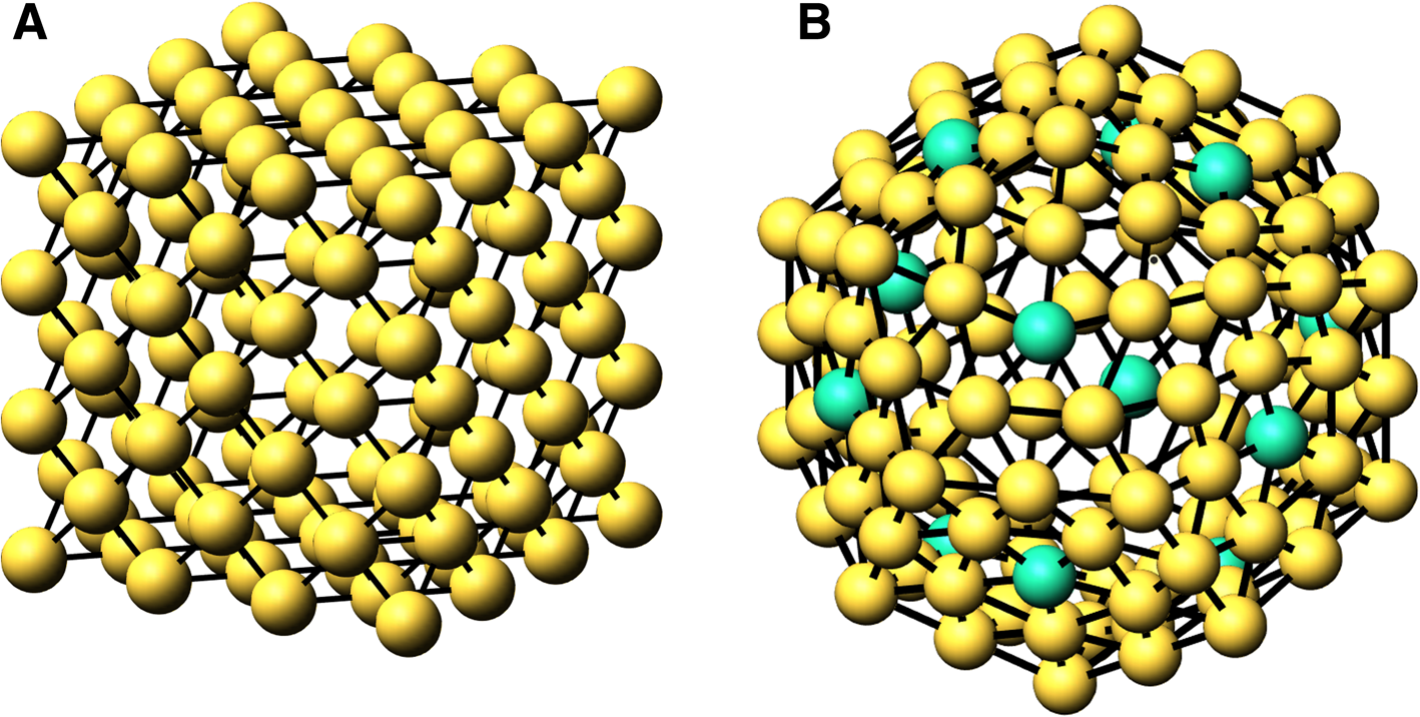
Shells of atoms for *n*=3 for A. fcc cube and *n*=2 B. dodecahedron. In B, the green atoms (12) refer to *cn*=5 within the shell

This holds if all the non-surface vertices have coordination larger than *cn*_*s*_, which is true for all clusters, but note the discrepancy for the dodecahedra below. We determine the $\phantom {\dot {i}\!}N_{{cn}_{i}}(n)$ by counting the columns of the adjacency matrix whose sum is *cn*_*i*_. Note that our cluster coordinate algorithm is built by shells, so that each subsequent shell contains all the previous lower values of *n*. In Fig. 1, we illustrate the shells of the clusters for an fcc cube and a dodecahedron. In addition, the number of bonds in the cluster is

5 $$ N_{B}(n) = \frac{1}{2}\sum_{{cn}_{i}=1}^{{cn}_{M}}{{cn}_{i}\cdot N_{{cn}_{i}}(n)},   $$

where *N*_*B*_(*n*) is the number of bonds and *cn*_*M*_ is the maximum coordination. The factor of 1/2 comes about because of the pairwise nearest neighbor bonding. This magic relationship appears not to have been considered in previous publications, with the exception of a few clusters examined in [4]. We also comment that Teo and Sloane have derived the total number of atoms, surface atoms, and interior atoms for clusters as follows [2]:

6 $$ N_{T}(n) = \alpha n^{3}+\frac{1}{2}\beta n^{2}+\gamma n+1~~n\ge{0}   $$

where *N*_*T*_(*n*) is the total number of atoms, and

7 $$ \alpha = C/6   $$

where *C* is the number of tetrahedral cells into which the polyhedron is divided, and

8 $$ \beta = 1/2F_{s}   $$

where *F*_*s*_ is the number of triangular faces on the surface, and

9 $$ \gamma = F_{s}/4+V_{i}+1-C/6   $$

where *V*_*i*_ is the number of vertices in the interior. They also show that

10 $$ N_{S}(n) = \beta n^{2}+2~~n\ge{1};~~N_{S}(0) = 1   $$

and

11 $$ N_{I}(n) = N_{T}(n) - N_{S}(n),   $$

where *N*_*I*_(*n*) is the number of interior atoms. This information (Eq. (11)) is contained in the adjacency matrix, as well as Eqs. (3, 4, 5). These equations are a check of the results from the adjacency matrix data. For centered polyhedra, we also have

12 $$ N_{I}(n) = N_{T}(n-1),   $$

and from Eq. (11), we have

13 $$ N_{T}(n)=N_{S}(n)+N_{S}(n-1)+... +N_{S}(1)+N_{S}(0).   $$

From these equations, we can derive the magic formulas for each of the clusters as follows. After computing the topological (0,1)-adjacency matrix **A** for a cluster with *n* shells as described, we know that its size *N*=*N*_*T*_(*n*) indicates the total number of atoms. The sum of the entries in column *i* gives the number of bonds *cn*_*i*_(*n*) for atom *i* and counting the the number of column sums equal to *cn*_*i*_(*n*) gives obviously $\phantom {\dot {i}\!}N_{{cn}_{i}}(n)$. Since we know that these depend on *n* as a polynomial of degree at most 3, we can compute *N*_*T*_(*n*) and *cn*_*i*_(*n*) for 4 consecutive values of *n*, say *n*=*n*_0_+*j*, *j*=0,1,2,3. A simple interpolating polynomial will then give the polynomial coefficients. It has to be verified that by increasing *n*_0_, which is usually equal to 1, the formulas do not change. If the formulas become stable from *n*_0_ on, then they hold for all *n*≥*n*_0_. In some cases, the polynomial relation only holds for the even *n* values or the odd ones. For example, for the fcc rhombic dodecahedron (Table 1), the successive shells have eight atoms with coordination 3 when *n*≥2 is even, and none if *n* is odd. In such cases different polynomial relations will hold for *n* even and *n* odd, but the data are used for *n*=*n*_0_+*j*, *j*=0,2,4,6 with *n*_0_ odd (e.g., *n*_0_=1) or *n*_0_ even (*n*_0_=2). To get the exact rational coefficients, one needs to solve the Vandermonde system for the coefficients in exact arithmetic using MATLAB’s symbolic toolbox. This is how the Tables 2, 3, 4, 5, 1, 6, 7, 8, 9, 10, 11, 12, 13, 14, 15, 16, 17, 18, and 19 are computed. In the next section, we determine magic formulas for *N*_*T*_(*n*), *N*_*B*_(*n*), and for $\phantom {\dot {i}\!}N_{{cn}_{i}}(n)$ according to the proscribed recipe.

**Table 1:** Magic formulas for the rhombic dodecahedron

	fcc rhombic dodecahedron *n*=4	
	Atoms	8*n*^3^+6*n*^2^+2*n*+3, *n*≥1 odd
		8*n*^3^+6*n*^2^+2*n*+1, *n*≥2 even
	Bonds	48*n*^3^+12, *n*≥1 odd;
		48*n*^3^,*n*≥2 even
	*cn*=3	8, *n* ≥2 even
	*cn*=4	6, *n* ≥1
	*cn*=5	12*n*−12, *n* ≥1 odd
		12*n*−24, *n* ≥2 even
	*cn*=7	12*n*^2^−12*n*+12, *n* ≥1
	*cn*=10	12*n*−12, *n* ≥1 odd
		12*n*, *n* ≥2 even
	*cn*=11	12*n*^2^−24*n*+12, *n* ≥1 odd
		12*n*^2^−24*n*, *n* ≥2, even
	*cn*=12	8*n*^3^−18*n*^2^+14*n*−3, *n* ≥1 odd
		8*n*^3^−18*n*^2^+14*n*−1,*n*≥2 even

**Table 2:** Magic formulas for the fcc cube

	fcc cube *n*=2	
	Atoms	4*n*^3^+6*n*^2^+3*n*+1, *n*≥1
	Bonds	24*n*^3^+12*n*^2^, *n*≥1
	*cn*=3	8, *n*≥1
	*cn*=5	12*n*−12, *n*≥1
	*cn*=8	12*n*^2^−12*n*+6, *n*≥1
	*cn*=12	4*n*^3^−6*n*^2^+3*n*−1, *n*≥1

**Table 3:** Magic formulas for the octahedron

	fcc octahedron *n*=6	
	Atoms	$\frac {2}{3}n^{3} + 2n^{2} + \frac {7}{3}n+1,~n\ge 1$
	Bonds	4*n*^3^+6*n*^2^+2*n*, *n*≥0
	*cn*=4	6, *n*≥1
	*cn*=7	12*n*−12, *n*≥1
	*cn*=9	4*n*^2^−12*n*+8, *n*≥1
	*cn*=12	$\frac {2}{3}n^{3}-2n^{2}+\frac {7}{3}n-1,~n\ge 1$

**Table 4:** Magic formulas for the cuboctahedron

	fcc cuboctahedron *n*=4	
	Atoms	$\frac {10}{3}n^{3} + 5n^{2} + \frac {11}{3}n+1,~n\ge 1$
	Bonds	20*n*^3^+12*n*^2^+4*n*, *n*≥0
	*cn*=5	12, *n*≥1
	*cn*=7	24*n*−24, *n*≥1
	*cn*=8	6*n*^2^−12*n*+6, *n*≥1
	*cn*=9	4*n*^2^−12*n*+8, *n*≥1
	*cn*=12	$\frac {10}{3}n^{3}-5n^{2}+\frac {11}{3}n-1,~n\ge 1$

**Table 5:** Magic formulas for the truncated octahedron

	fcc truncated octahedron *n*=2	
	Atoms	16*n*^3^+15*n*^2^+6*n*+1, *n*≥1
	Bonds	96*n*^3^+42*n*^2^+6*n*, *n*≥0
	*cn*=6	24, *n*≥1
	*cn*=7	36*n*−36, *n*≥1
	*cn*=8	6*n*^2^−12*n*+6, *n*≥1
	*cn*=9	24*n*^2^−24*n*+8, *n*≥1
	*cn*=12	16*n*^3^−15*n*^2^+6*n*−1, *n*≥1

**Table 6:** Magic formulas for the bcc cube

	bcc cube	
	Atoms	2*n*^3^+3*n*^2^+3*n*+1, *n*≥1
	Bonds	14*n*^3^+3*n*^2^+3*n*, *n*≥1
	*cn*=4	8, *n*≥1
	*cn*=6	12*n*−12, *n*≥1
	*cn*=8	1, *n*=1; 0, *n*≠1
	*cn*=9	6*n*^2^−12*n*+6, *n*≥1
	*cn*=11	8, *n*≥2
	*cn*=12	12*n*−24, *n*≥2
	*cn*=13	6*n*^2^−24*n*+24, *n*≥2
	*cn*=14	2*n*^3^−9*n*^2^+15*n*−9, *n*≥2

**Table 7:** Magic formulas for the bcc octahedron

	bcc octahedron *n*=4	
	Atoms	$\frac {8}{3}n^{3}+6n^{2}+\frac {16}{3}n+1,~n\ge 1$
	Bonds	$\frac {56}{3}n^{3}+18n^{2}+\frac {40}{3}n,~n\ge 0$
	*cn*=5	6, *n*≥1
	*cn*=7	4*n*^2^+4*n*, *n*≥0
	*cn*=8	12*n*−12, *n*≥1
	*cn*=10	4*n*^2^−12*n*+8, *n*≥1
	*cn*=13	4*n*^2^−4*n*, *n*≥1
	*cn*=14	$\frac {8}{3}n^{3}-6n^{2}+\frac {16}{3}n-1,~n\ge {1}$

**Table 8:** Magic formulas for the bcc truncated octahedron

	bcc truncated octahedron *n*=4	
	Atoms	$8n^{3}+\frac {9}{2}n^{2} +\frac {5}{2},~n\ge 1$ odd
		$8n^{3}+\frac {9}{2}n^{2} +3n+ 1,~n\ge 2$ even
	Bonds	$56n^{3}-\frac {27}{2}n^{2}-6n+\frac {27}{2},~n\ge 1$ odd
		$56n^{3}+\frac {27}{2}n^{2}+3n,~n\ge 2$ even
	*cn*=4	0, *n*≥1 odd
		6*n*+12, *n*≥2 even
	*cn*=6	24, *n*≥3 odd
		12*n*−24, *n*≥2 even
	*cn*=7	6*n*^2^+12*n*−34,*n*≥3 odd
		6*n*^2^−12*n*+8,*n*≥2 even
	*cn*=8	6*n*−6, *n*≥1 odd; 0, *n* even
	*cn*=9	3*n*^2^−12*n*+15, *n*≥3 odd
		3*n*^2^−6*n*+6, *n*≥2 even
	*cn*=10	6*n*^2^−12*n*+6, *n*≥1 odd
		6*n*^2^, *n*≥2 even
	*cn*=12	12*n*−12, *n*≥1 odd
		6*n*, *n*≥2 even
	*cn*=13	9*n*^2^−24*n*+15, *n*≥1 odd
		9*n*^2^−18*n*, *n*≥2 even
	*cn*=14	$8n^{3}-\frac {39}{2}n^{2}+18n-\frac {11}{2},~n\ge {1}$ odd
		$8n^{3}-\frac {39}{2}n^{2}+15n-1,~n\ge {2}$ even

**Table 9:** Magic formulas for the bcc cuboctahedron

	bcc cuboctahedron *n*=3	
	Atoms	$\frac {5}{3}n^{3}+7n^{2}+\frac {34}{3}n+7,~n\ge 1$ odd
		$\frac {5}{3}n^{3}+7n^{2}+\frac {25}{3}n+1,~n\ge 2$ even
	Bonds	$\frac {35}{3}n^{3}+34n^{2}+\frac {112}{3}n+15,n\ge 1$ odd
		$\frac {35}{3}n^{3}+34n^{2}+\frac {67}{3}n,~n\ge 2$ even
	*cn*=4	12, *n*≥1 odd; 0, *n* even
	*cn*=6	12*n*−12, *n*≥1 odd; 0, *n* even
	*cn*=7	*n*^2^−4*n*+3, *n*≥1 odd
		*n*^2^+14*n*, *n*≥2 even
	*cn*=9	3*n*^2^+3, *n*≥1 odd
		3*n*^2^−6*n*, *n*≥2 even
	*cn*=10	*n*^2^+4*n*+3, *n*≥1, odd
		*n*^2^−2*n*+12, *n*≥2, even
	*cn*=12	12*n*−24, *n*≥2 even; 0, *n* odd
	*cn*=13	4*n*^2^−4, *n*≥3 odd
		4*n*^2^−12*n*+14, *n*≥2 even
	*cn*=14	$\frac {5}{3}n^{3}-2n^{2}-\frac {2}{3}n+2,~n\ge {1}$ odd
		$\frac {5}{3}n^{3}-2n^{2}+\frac {7}{3}n-1,~n\ge {2}$ even

**Table 10:** Magic formulas for the bcc rhombic dodecahedron

	bcc rhombic dodecahedron *n*=3	
	Atoms	4*n*^3^+6*n*^2^+4*n*+1, *n*≥1
	Bonds	28*n*^3^+18*n*^2^+4*n*, *n*≥0
	*cn*=5	6, *n* ≥1
	*cn*=7	8, *n* ≥1
	*cn*=8	24*n*−24, *n* ≥1
	*cn*=10	12*n*^2^−24*n*+12, *n* ≥2
	*cn*=14	4*n*^3^−6*n*^2^+4*n*−1, *n* ≥1

**Table 11:** Magic formulas for the hexagonal bipyramid

	Hexagonal bipyramid *n*=4	
	Atoms	4*n*^3^+6*n*^2^+4*n*+1, *n*≥1
	Bonds	24*n*^3^+15*n*^2^+3*n*, *n*≥0
	*cn*=3	2, *n*≥1
	*cn*=5	6, *n*≥1
	*cn*=6	3*n*+3, *n*≥1
	*cn*=7	24*n*−24, *n*≥1
	*cn*=8	6*n*^2^−15*n*+9, *n*≥1
	*cn*=9	6*n*^2^−12*n*+6, *n*≥1
	*cn*=12	4*n*^3^−6*n*^2^+4*n*−1, *n*≥1

**Table 12:** Magic formulas for the truncated hexagonal bipyramid

	Truncated hexagonal bipyramid *n*=4	
	Atoms	$\frac {7}{2}n^{3} + \frac {21}{4}n^{2} + \frac {7}{2}n + \frac {3}{4},~n\ge 3$ odd
		$\frac {7}{2}n^{3} + \frac {21}{4}n^{2} + \frac {7}{2}n + 1,~n\ge 2$ even
	Bonds	$21n^{3} + \frac {27}{2}n^{2} + 3n - \frac {3}{2},~n\ge 3$ odd
		$21n^{3} + \frac {27}{2}n^{2} + 3n,~n\ge 2$ even
	*cn*=5	6, *n*≥2
	*cn*=6	3*n*+9, *n*≥1
	*cn*=7	18*n*−24, *n*≥1
	*cn*=8	$\frac {9}{2}n^{2} - 9n + \frac {9}{2},~n \ge {3}$, odd
		$\frac {9}{2}n^{2} - 9n + 3,~n \ge {2}$, even
	*cn*=9	6*n*^2^−12*n*+6, *n*≥3, odd
		6*n*^2^−12*n*+8, *n*≥2, even
	*cn*=12	$\frac {7}{2}n^{3} - \frac {21}{4}n^{2} + \frac {7}{2}n -\frac {3}{4},~n\ge 3$ odd
		$\frac {7}{2}n^{3} - \frac {21}{4}n^{2} + \frac {7}{2}n -1,~n\ge 2$ even

**Table 13:** Magic formulas for the icosahedron

	Icosahedron *n*=4	
	Atoms	$\frac {10}{3}n^{3} + 5n^{2} + \frac {11}{3}n + 1,~n\ge 1$
	Bonds	20*n*^3^+15*n*^2^+7*n*, *n*≥1
	*cn*=6	12, *n*≥1
	*cn*=8	30*n*−30, *n*≥1
	*cn*=9	10*n*^2^−30*n*+20, *n*≥1
	*cn*=12	$\frac {10}{3}n^{3} - 5n^{2} + \frac {11}{3}n -1,~n\ge 1$

**Table 14:** Magic formulas for the dodecahedron

	Dodecahedron *n*=3	
	Atoms	10*n*^3^+15*n*^2^+7*n*+1, *n*≥1
	Bonds	40*n*^3^+45*n*^2^+17*n*, *n*≥0
	Surface atoms	30*n*^2^+2, *n*≥1
	*cn*=6	30*n*+2, *n*≥1
	*cn*=7 Bulk	12*n*−12, *n*≥2
	*cn*=7 Surface	30*n*^2^−30*n*, *n*≥2
	*cn*=8	10*n*^3^−15*n*^2^−25*n*+30, *n*≥1
	*cn*=9	20*n*−20, *n*≥1
	*cn*=12	1, *n*≥1

**Table 15:** Magic formulas for the fcc tetrahedron

	fcc tetrahedron *n*=6	
	Atoms	$\frac {1}{6}n^{3} + n^{2} + \frac {11}{6}n + 1,~n\ge 1$
	Bonds	*n*^3^+3*n*^2^+2*n*, *n*≥1
	*cn*=3	4, *n*≥1
	*cn*=6	6*n*−6, *n*≥1
	*cn*=9	2*n*^2^−6*n*+4, *n*≥1
	*cn*=12	$\frac {1}{6}n^{3} - n^{2} + \frac {11}{6}n -1,~n \ge {1}$

**Table 16:** Magic formulas for the bcc tetrahedron

	bcc tetrahedron *n*=4	
	Atoms	$\frac {1}{3}n^{3} + \frac {3}{2}n^{2} + \frac {13}{6}n + 1,~n\ge 1$
	Bonds	$\frac {2}{3}n^{3} + 2n^{2} + \frac {4}{3}n,~n\ge 1$
	*cn*=1	4, *n*≥1
	*cn*=2	6*n*−6, *n*≥1
	*cn*=3	2*n*^2^−6*n*+4, *n*≥1
	*cn*=4	$\frac {1}{3}n^{3} - \frac {1}{2}n^{2} + \frac {13}{6}n -1,~n\ge 1$

**Table 17:** Magic formulas for the diamond cubic

	Diamond cubic *n*=3	
	Atoms	8*n*^3^+6*n*^2^+3*n*−3,*n*≥1
	Bonds	16*n*^3^
	*cn*=1	12*n*−8,*n*≥1
	*cn*=2	12*n*^2^−12*n*+6,*n*≥1
	*cn*=4	8*n*^3^−6*n*^2^+3*n*−1,*n*≥1

**Table 18:** Magic formulas for the simple cubic

	Simple cube *n*=2	
	Atoms	8*n*^3^
	Bonds	24*n*^3^−12*n*^2^
	*cn*=3	8
	*cn*=4	24*n*−24, *n*≥2
	*cn*=5	24*n*^2^−48*n*+24, *n*≥2
	*cn*=6	8*n*^3^−24*n*^2^+24*n*−8, *n*≥2

**Table 19:** Magic formulas for the decahedron

	Decahedron *n*=4	
	Atoms	$\frac {5}{6}n^{3} + \frac {5}{2}n^{2}+\frac {8}{3}n +1$
	Bonds	$5n^{3} + \frac {15}{2}n^{2} + \frac {7}{2}n$
	*cn*=4	5, *n*≥1
	*cn*=6	5*n*−3, *n*≥1
	*cn*=8	10*n*−10, *n*≥1
	*cn*=9	5*n*^2^−15*n*+10, *n*≥1
	*cn*=12	$\frac {5}{6}n^{3} - \frac {5}{2}n^{2} + \frac {8}{3}n -1,~n\ge 1$

The dispersion (fraction exposed, FE) of the surface atoms is defined as:

14 $$ \text{FE} = \frac{N_{S}}{N_{T}} \cdot 100\%   $$

where *N*_*S*_ is the number of surface atoms, and *N*_*T*_ is the total number of atoms [9]. We can compare dissimilar clusters by defining the relative cluster size as:

15 $$ d_{rel} = b(N_{T})^{1/3};~~b = d_{at}^{-1}\cdot \left(\frac{6V_{u}}{\pi n_{u}}\right)^{1/3}   $$

where *d*_*at*_ is the covalent atomic diameter, *V*_*u*_ is the volume of the unit cell, and *n*_*u*_ is the number of atoms in the unit cell. The crystal structure constant *b* equals 1.105 for fcc and hcp clusters, 1.137 for bcc clusters [1], 1.488 for simple cubic clusters, and 1.517 for diamond cubic clusters. As is shown above, the formula for FE is a ratio of a quadratic to a cubic for the clusters and can be modeled by a power law curve fit versus *d*_*rel*_. The variable *d*_*rel*_ allows us to compare different clusters to one another without regard to the crystal structure. For some of the Platonic clusters, where there is no crystal unit cell, we use $N_{T}^{1/3}$ as the variable.

## Results and Discussion

The study of the size and shape of metal nanoclusters has evolved since its infancy two decades ago. Table 20 shows some relevant progress as of 2018.

**Table 20:** Shape-dependent synthesis for nanoclusters circa 2018

In the Table, we list primarily transition metals, not alloys or compounds, with the exception of the truncated hexagonal bipyramid, where only Fe_2_O_3_ was found. There has been more synthesis of gold clusters than any other element, due to its properties and stability. In the subsections which follow, we limit our discussion to specific topics related to magic formulas and types of clusters.

### FCC Clusters

Eight of the transition metals crystallize in the fcc structure, see Table 21 below, including the plasmonic noble metals and important catalytically active elements. The vast majority of nanocluster synthesis has been with these elements. References of the synthesis of the fcc elements with various shapes and sizes is given in Table 21.

**Table 21:** Structure of the transition metals [15]

Sc	Ti	V	Cr	Mn	Fe	Co	Ni	Cu	Zn
hcp	hcp	bcc	bcc	cubic	bcc	hcp	fcc [16]	fcc [17]	hcp
Y	Zr	Nb	Mo	Tc	Ru	Rh	Pd	Ag	Cd
hcp	hcp	bcc	bcc	hcp	hcp	fcc [18]	fcc [19]	fcc [20]	hcp
La	Hf	Ta	W	Re	Os	Ir	Pt	Au	Hg
hex	hcp	bcc	bcc	hcp	hcp	fcc [21]	fcc [22]	fcc [23]	rhomb

Alloys of these elements are also of interest, but references of these are too numerous to be cited here. Frequently, the common shapes synthesized are cubes, octahedra, cuboctahedra, and icosahedra. Typically, clusters with (111) facets are easier to synthesize, since the (111) surface usually has a lower energy than the (100) surface [7]. We find for the fcc rhombic dodecahedron that there exist even and odd formulas. These agree with those in [1], if one replaces the “*n*” in our even formulas by 2(*m*−1). The formulas for fcc cuboctahedra listed in [24] produce the same magic numbers as ours but are shifted by 1 since they number shells as *n*=1,2,… and we use the numbering *n*=0,1,…. Our magic formulas agree with those in [2, 4], and in deference to the earlier published work, and in maintaining continuity of the mathematics, we use the [2, 4] notation. The 5 fcc cluster shapes and their associated magic formulas appear below.

### BCC Clusters

Seven of the transition metals in the periodic table have the bcc structure, see Table 21. Of the magnetic elements Fe, Co, and Ni, only iron is bcc. Nanocubes of iron appear to be the only bcc cluster shape synthesized so far [25]. Although the bulk structure of iron is bcc, fcc nanoclusters have been synthesized [26]. This reference also analyzes the thermodynamic stability of the clusters. Here we present 5 bcc cluster shapes and their associated magic formulas.

### HCP Clusters

Twelve transition metals have the hcp structure, see Table 21. However, many of these oxidize, or lack compelling scientific interest to be synthesized. With regard to the hexagonal bipyramidal cluster shape in Table 11, gold clusters have been synthesized [27]. The related truncated hexagonal bipyramid seems only to have been formed by *α*−Fe_2_O_3_ [28].

### Platonic Clusters

The Platonic solids have been known since the ancient Greeks. They include the cube, tetrahedron, octahedron, icosahedron, and dodecahedron. In previous tables, we have listed magic formulas for fcc and bcc cubes and octahedra. Here we list the formulas for the icosahedron, dodecahedron, tetrahedron, and body-centered tetrahedron. As previously mentioned in the “Methods” section, the dodecahedron is unique for the clusters analyzed here, in that *cn*_*s*_=7 refers to both surface and bulk atoms. We showed in Fig. 1b that the outer shell contains both fivefold and sixfold coordinated atoms. When a shell becomes internal, those five- and sixfold coordinated atoms become seven- and eightfold coordinated with bonds to a shell on either side. Also, the sixfold coordinated outside shell atoms are sevenfold coordinated by bonding to the shell inside. Thus there are sevenfold surface and bulk coordinated atoms for the dodecahedron. Each shell in the structure has 12 fivefold shell atoms, which produce 12*n*−12 bulk sevenfold coordinated atoms. The rest of the sevenfold coordination are surface atoms.

Gold nanoclusters have been shown to take the Platonic shapes [29]. This reference includes the cube, tetrahedron, octahedron, and icosahedron. Later, the golden dodecahedron nanocluster was also synthesized [30]. Here, we show both the regular tetrahedron, which is “fcc-like” in that *c*_*M*_=12 as in fcc structures, and the body-centered tetrahedron in Table 16, where the green atoms have single bonds. The Platonic magic formulas are presented below.

### Diamond Cubic, Simple Cubic, and Decahedron Clusters

The elements silicon and germanium have the diamond cubic lattice, as well as the diamond allotrope of carbon. In particular, hydrogen-terminated silicon has received recent interest. The (100) hydrogen-terminated surface, leading to cubic shapes in clusters, has been determined to have the lowest energy [31]. The synthesis of Si-H nanocubes of 8−15 nm in size has been achieved [32]. Table 17 shows a diagram of the hydrogen-terminated Si-H clusters, with single-bonded hydrogen atoms in green. If a nanocluster takes the diamond cubic shape, there will be single dangling bonds, which need to be passivated to help maintain the structure. Looking at the magic formulas, we suggest the composition of such Si-H clusters is $\phantom {\dot {i}\!}\text {Si}_{8n^{3}+6n^{2}-9n+5}\text {H}_{12n-8}$, where *n* is the number of shells in the cluster. Such semiconductor quantum dots may be of interest for optical properties, and the variation in band-gap with the size of hydrogen-terminated clusters has been determined to be inversely proportional to the cluster size [33].

The simple cubic lattice structure has previously been analyzed by others [4], although without the detail we provide. We have previously studied the *d*-dimensional hypercube forms [14]. Polonium is the only element which takes the simple cubic structure. It is radioactive, which may lead to specialized applications. Here we present the diamond cubic, simple cubic, and decahedral cluster magic formulas.

### Magic Topological Formulas

Measured structural complexity in crystals can give us an idea of the simplicity or complexity of the structure and the proper use can rank relevant structures. For such rankings, it is helpful to consider the graphical description of the crystal lattice, as mentioned in the “Methods” section. The topological complexity for crystal structures is measured by the vertex-degree distribution of the graph, *I*_*vd*_ [34], using the software ToposPro, version 5.3.2.2 [35]:

16 $$ I_{vd} = \sum_{i=1}^{v}a_{i} \cdot {\text{log}_{2}}\ {a_{i}}   $$

where *a*_*i*_ is the degree (coordination) of the *i*th vertex and summation proceeds along all *v* vertices, of the quotient graph. This parameter uses an infinite crystal as opposed to the clusters we have been considering, but is useful to measure the relative complexity of different crystal structures. Thus, the higher the number, or the more information content in the graph, the more complex it is. In Table 22, we show values of *I*_*vd*_ obtained from ToposPro derived from cif files for crystal structures in the Crystallographic Open Database. Polonium is the only element that crystallizes in the simple cubic structure and the value is zero, i.e., the quotient graph has one vertex and zero edges, in agreement with what we expect, that the simple cubic structure is indeed the least complex structure. The salt, NaCl, is also shown, with two elements in the simple cubic structure, along with silicon in the diamond cubic, gold in fcc, iron in bcc, and cobalt in hcp structures. We mention that another complexity measure related to the Shannon entropy [34] is not useful because this measure for all the elements is identically zero.

**Table 22:** Topological complexity

Structure	*I*_*vd*_
Po simple cubic	0.000
Si diamond cubic	16.000
NaCl	31.020
Au fcc	43.020
Fe bcc	53.303
Co hcp	86.039

A similar method as described in the “Methods” section to determine magic formulas can be applied for the magic formulas describing the topological indices. Only here, the degrees of the polynomials are 7, 8, or 9, so their values for at least 10 consecutive *n*-values need to be computed. Then an interpolation problem of a higher degree gives the result. Since solving a linear system of size 10×10 with the symbolic toolbox requires some time, all the coefficients for the topological indices can be computed simultaneously using multiple right-hand sides to get the coefficients of all the polynomials.

Magic formulas for the topological indices are detailed in Tables 23, 24, and 25. The four indices we analyze depend only on *n*, the number of shells in the cluster. Looking at the results, the simple cubic lattice as the least complex structure, also has the “simplest” formulas. In spite of our efforts, we are unable to solve for the Szeged index of bcc cubes. No stable solution was found. In general, fcc structures are easier to solve for topological formulas. We were not able to solve any hcp structures and only a few bcc structures. This may be related to the topological complexity as the fcc lattice is simpler than the bcc or hcp, see Table 22. Within the tables, we provide formulas for the cuboctahedron, icosahedron, and decahedron. We previously [12] provided tables of numeric data for these indices, with the caveat that the cuboctahedron in [12] had different magic numbers. Here we see that the tabulated data may be succinctly summarized as magic formulas. Also the degree of the polynomial of the index follows the rules from 3D space [14]. Some topological indices for the Platonic solids have previously been published [36]. From this reference, we verify the Wiener index for all five solids for *n*=1. The Wiener index for rows of unit cells of the fcc lattice has been studied [37], but our results cannot be compared since we study clusters.

**Table 23:** Magic topological formulas for clusters

Simple cubic	
Wiener	64*n*^7^−16*n*^5^
Reverse Wiener	128*n*^7^−96*n*^6^+16*n*^5^−24*n*^4^+12*n*^3^
HyperWiener	$\frac {224}{3}n^{8} + 32n^{7} - \frac {88}{3}n^{6} - 8n^{5} + \frac {8}{3}n^{4}$
Szeged	256*n*^9^−64*n*^7^
fcc cube	
Wiener	$\frac {956}{105}n^{7} + \frac {478}{15}n^{6} + \frac {1357}{30}n^{5} + \frac {110}{3}n^{4} + \frac {589}{30}n^{3}+\frac {97}{15}n^{2}+\frac {36}{35}n$
Reverse Wiener	$\frac {1564}{105}n^{7} + \frac {602}{15}n^{6} + \frac {1343}{30}n^{5} + \frac {70}{3}n^{4} + \frac {43}{15}n^{3}-\frac {59}{30}n^{2}-\frac {36}{35}n$
HyperWiener	$\frac {59}{10}n^{8}+\frac {2956}{105}n^{7} + \frac {1089}{20}n^{6} + \frac {701}{12}n^{5} + \frac {817}{20}n^{4} + \frac {1153}{60}n^{3}+\frac {53}{10}n^{2}+\frac {5}{7}n$
Szeged	$\frac {14822}{945}n^{9}+\frac {2099}{35}n^{8} + \frac {30781}{315}n^{7}+\frac {941}{10}n^{6} + \frac {1073}{18}n^{5} + \frac {251}{10}n^{4} + \frac {12629}{1890}n^{3}+\frac {29}{35}n^{2}+\frac {32}{105}n$
bcc cube	
Wiener	$\frac {12}{7}n^{7} + 6n^{6} + \frac {59}{5}n^{5} + \frac {29}{2}n^{4} + \frac {34}{3}n^{3}+\frac {11}{2}n^{2}+\frac {121}{105}n$
Reverse Wiener	$\frac {16}{7}n^{7} + 6n^{6} + \frac {46}{5}n^{5} + \frac {11}{2}n^{4} + \frac {2}{3}n^{3}-\frac {5}{2}n^{2}-\frac {121}{105}n$
HyperWiener	$\frac {71}{84}n^{8}+\frac {89}{21}n^{7} + \frac {53}{5}n^{6} + \frac {253}{15}n^{5} + \frac {421}{24}n^{4} + \frac {143}{12}n^{3}+\frac {4211}{840}n^{2}+\frac {137}{140}n$
Szeged	NA
fcc octahedron	
Wiener	$\frac {59}{420}n^{7} + \frac {59}{60}n^{6} + \frac {179}{60}n^{5} + \frac {61}{12}n^{4} + \frac {77}{15}n^{3}+\frac {44}{15}n^{2}+\frac {26}{35}n$
Reverse Wiener	$\frac {383}{1260}n^{7} + \frac {101}{60}n^{6} + \frac {743}{180}n^{5} + \frac {59}{12}n^{4} + \frac {104}{45}n^{3}-\frac {3}{5}n^{2}-\frac {26}{35}n$
HyperWiener	$\frac {173}{3360}n^{8}+\frac {27}{56}n^{7} + \frac {463}{240}n^{6} + \frac {87}{20}n^{5} + \frac {2891}{480}n^{4} + \frac {41}{8}n^{3}+\frac {699}{280}n^{2}+\frac {19}{35}n$
Szeged	$\frac {397}{5040}n^{9}+\frac {397}{560}n^{8} + \frac {347}{120}n^{7}+\frac {841}{120}n^{6} + \frac {891}{80}n^{5} + \frac {2897}{240}n^{4} + \frac {2801}{315}n^{3}+\frac {1769}{420}n^{2}+1n$
fcc cuboctahedron	
Wiener	$\frac {204}{35}n^{7} + \frac {102}{5}n^{6} + \frac {168}{5}n^{5} + 33n^{4} + \frac {98}{5}n^{3}+\frac {33}{5}n^{2}+\frac {34}{35}n$
Reverse Wiener	$\frac {1664}{315}n^{7} + \frac {194}{15}n^{6} + \frac {713}{45}n^{5} + 7n^{4} - \frac {52}{45}n^{3}-\frac {44}{15}n^{2}-\frac {34}{35}n$
HyperWiener	$\frac {487}{140}n^{8}+\frac {589}{35}n^{7} + \frac {433}{12}n^{6} + \frac {183}{4}n^{5} + \frac {548}{15}n^{4} + \frac {357}{20}n^{3}+\frac {103}{21}n^{2}+\frac {4}{7}n$
Szeged	$\frac {68867}{7560}n^{9}+\frac {12589}{336}n^{8} + \frac {3269}{45}n^{7}+\frac {10403}{120}n^{6} + \frac {23759}{360}n^{5} + \frac {1475}{48}n^{4} + \frac {30929}{3780}n^{3}+\frac {467}{420}n^{2}+\frac {1}{15}n$

**Table 24:** Magic topological formulas for clusters, continued

fcc truncated octahedron	
Wiener	$\frac {31813}{140}n^{7} + \frac {29741}{60}n^{6} + \frac {1925}{4}n^{5} + \frac {3259}{12}n^{4} + \frac {469}{5}n^{3}+\frac {281}{15}n^{2}+\frac {12}{7}n$
Reverse Wiener	$\frac {39867}{140}n^{7} + \frac {27859}{60}n^{6} + \frac {1411}{4}n^{5} + \frac {1445}{12}n^{4} + \frac {41}{5}n^{3}-\frac {101}{15}n^{2}-\frac {12}{7}n$
HyperWiener	$\frac {258927}{1120}n^{8}+\frac {115583}{168}n^{7} + \frac {211547}{240}n^{6} + \frac {19453}{30}n^{5} + \frac {144307}{480}n^{4} + \frac {2099}{24}n^{3}+\frac {12373}{840}n^{2}+\frac {39}{35}n$
Szeged	$\frac {1120559}{1080}n^{9}+\frac {598387}{210}n^{8} + \frac {640481}{180}n^{7}+\frac {80023}{30}n^{6} + \frac {478073}{360}n^{5} + \frac {6677}{15}n^{4} + \frac {13388}{135}n^{3}+\frac {489}{35}n^{2}+\frac {16}{15}n$
bcc rhombic dodecahedron	
Wiener	$\frac {293}{35}n^{7} + \frac {293}{10}n^{6} + \frac {93}{2}n^{5} + 43n^{4} + \frac {721}{30}n^{3}+\frac {77}{10}n^{2}+\frac {23}{21}n$
Reverse Wiener	$\frac {267}{35}n^{7} + \frac {187}{10}n^{6} + \frac {43}{2}n^{5} + 9n^{4} - \frac {61}{30}n^{3}-\frac {37}{10}n^{2}-\frac {23}{21}n$
HyperWiener	$\frac {4187}{840}n^{8}+\frac {2533}{105}n^{7} + \frac {1011}{20}n^{6} + \frac {367}{6}n^{5} + \frac {5549}{120}n^{4} + \frac {647}{30}n^{3}+\frac {601}{105}n^{2}+\frac {9}{14}n$
Szeged	$\frac {29447}{1890}n^{9}+\frac {110993}{1680}n^{8} + \frac {158141}{1260}n^{7}+\frac {16897}{120}n^{6} + \frac {18109}{180}n^{5} + \frac {10931}{240}n^{4} + \frac {23221}{1890}n^{3}+\frac {221}{140}n^{2}+\frac {2}{105}n$
Icosahedron	
Wiener	$\frac {118}{21}n^{7} + \frac {59}{3}n^{6} + \frac {97}{3}n^{5} + \frac {95}{3}n^{4} + \frac {55}{3}n^{3}+\frac {17}{3}n^{2}+\frac {5}{7}n$
Reverse Wiener	$\frac {346}{63}n^{7} + \frac {41}{3}n^{6} + \frac {154}{9}n^{5} + \frac {25}{3}n^{4} + \frac {1}{9}n^{3}-2n^{2}-\frac {5}{7}n$
HyperWiener	$\frac {311}{96}n^{8}+\frac {883}{56}n^{7} + \frac {1627}{48}n^{6} + 43n^{5} + \frac {3263}{96}n^{4} + \frac {127}{8}n^{3}+\frac {31}{8}n^{2}+\frac {5}{14}n$
Szeged	$\frac {46049}{6048}n^{9}+\frac {46049}{1344}n^{8} + \frac {5521}{72}n^{7}+\frac {10415}{96}n^{6} + \frac {26417}{288}n^{5} + \frac {7303}{192}n^{4} + \frac {5735}{3024}n^{3}-\frac {1273}{336}n^{2}-\frac {11}{12}n$
Dodecahedron	
Wiener	$\frac {601}{7}n^{7} + \frac {601}{2}n^{6} + 416n^{5} + \frac {1155}{4}n^{4} + \frac {625}{6}n^{3}+\frac {71}{4}n^{2}+\frac {41}{42}n$
Reverse Wiener	$\frac {799}{7}n^{7} + \frac {599}{2}n^{6} + 314n^{5} + \frac {605}{4}n^{4} + \frac {143}{6}n^{3}-\frac {15}{4}n^{2}-\frac {41}{42}n$
HyperWiener	$\frac {2349}{28}n^{8}+\frac {757}{2}n^{7} + \frac {8203}{12}n^{6} + \frac {1267}{2}n^{5} + 321n^{4} + \frac {263}{3}n^{3}+\frac {242}{21}n^{2}+\frac {1}{3}n$
Szeged	$\frac {1623611}{6048}n^{9}+\frac {1623611}{1344}n^{8} + \frac {1231255}{504}n^{7}+\frac {93211}{32}n^{6} + \frac {630167}{288}n^{5} + \frac {64439}{64}n^{4} + \frac {806507}{3024}n^{3}+\frac {14869}{336}n^{2}+\frac {487}{84}n$
Decahedron	
Wiener	$\frac {121}{504}n^{7} + \frac {121}{72}n^{6} + \frac {355}{72}n^{5} + \frac {565}{72}n^{4} + \frac {257}{36}n^{3}+\frac {125}{36}n^{2}+\frac {29}{42}n$
Reverse Wiener	$\frac {229}{504}n^{7} + \frac {179}{72}n^{6} + \frac {415}{72}n^{5} + \frac {455}{72}n^{4} +\frac {89}{36}n^{3}-\frac {29}{36}n^{2}-\frac {29}{42}n$
HyperWiener	$\frac {7}{72}n^{8}+\frac {905}{1008}n^{7} + \frac {499}{144}n^{6} + \frac {1055}{144}n^{5} + \frac {1327}{144}n^{4} + \frac {493}{72}n^{3}+\frac {49}{18}n^{2}+\frac {3}{7}n$
Szeged	$\frac {9115}{72576}n^{9}+\frac {9115}{8064}n^{8} + \frac {54451}{12096}n^{7}+\frac {1999}{192}n^{6} + \frac {51751}{3456}n^{5} + \frac {4975}{384}n^{4} + \frac {26855}{4536}n^{3}+\frac {2021}{2016}n^{2}-\frac {11}{504}n$

**Table 25:** Magic topological formulas for clusters, continued

fcc tetrahedron	
Wiener	$\frac {1}{168}n^{7} + \frac {1}{12}n^{6} + \frac {7}{15}n^{5} + \frac {4}{3}n^{4} + \frac {49}{24}n^{3}+\frac {19}{12}n^{2}+\frac {17}{35}n$
Reverse Wiener	$\frac {1}{126}n^{7} + \frac {1}{12}n^{6} + \frac {61}{180}n^{5} + \frac {7}{12}n^{4} +\frac {5}{36}n^{3}-\frac {2}{3}n^{2}-\frac {17}{35}n$
HyperWiener	$\frac {1}{672}n^{8}+\frac {3}{112}n^{7} + \frac {47}{240}n^{6} + \frac {3}{4}n^{5} + \frac {155}{96}n^{4} + \frac {31}{16}n^{3}+\frac {499}{420}n^{2}+\frac {2}{7}n$
Szeged	$\frac {71}{60480}n^{9}+\frac {71}{3360}n^{8} + \frac {227}{1440}n^{7}+\frac {151}{240}n^{6} + \frac {4163}{2880}n^{5} + \frac {917}{480}n^{4} + \frac {20599}{15120}n^{3}+\frac {123}{280}n^{2}+\frac {1}{30}n$
bcc tetrahedron	
Wiener	$\frac {1}{21}n^{7} + \frac {1}{2}n^{6} + \frac {21}{10}n^{5} + \frac {9}{2}n^{4} + \frac {31}{6}n^{3}+3n^{2}+\frac {24}{35}n$
Reverse Wiener	$\frac {4}{63}n^{7} + \frac {1}{2}n^{6} + \frac {287}{180}n^{5} + \frac {7}{3}n^{4} + \frac {37}{36}n^{3}-\frac {5}{6}n^{2}-\frac {24}{35}n$
HyperWiener	$\frac {1}{42}n^{8}+\frac {13}{42}n^{7} + \frac {587}{360}n^{6} + \frac {179}{40}n^{5} + \frac {493}{72}n^{4} + \frac {139}{24}n^{3}+\frac {787}{315}n^{2}+\frac {89}{210}n$
Szeged	$\frac {1}{81}n^{9}+\frac {1}{6}n^{8} + \frac {176}{189}n^{7}+\frac {25}{9}n^{6} + \frac {641}{135}n^{5} + \frac {83}{18}n^{4} + \frac {188}{81}n^{3}+\frac {4}{9}n^{2}-\frac {4}{315}n$
Diamond cubic	
Wiener	$\frac {7648}{105}n^{7} + \frac {1912}{15}n^{6} + \frac {1792}{15}n^{5} - \frac {40}{3}n^{4} - \frac {374}{15}n^{3}-\frac {902}{15}n^{2}-\frac {48}{35}n+12$
Reverse Wiener	$\frac {12512}{105}n^{7} + \frac {1448}{15}n^{6} + \frac {548}{15}n^{5} - \frac {392}{3}n^{4} - \frac {811}{15}n^{3}+\frac {452}{15}n^{2}+\frac {2043}{35}n-24$
HyperWiener	$\frac {472}{5}n^{8}+\frac {23648}{105}n^{7} + \frac {3976}{15}n^{6} + \frac {842}{15}n^{5} - \frac {926}{15}n^{4} - \frac {1219}{15}n^{3}-\frac {971}{15}n^{2}+\frac {312}{35}n+18$
Szeged	$\frac {512}{3}n^{9}+\frac {5896}{21}n^{8} + 208n^{7}-\frac {1504}{5}n^{6} +\frac {503}{5}n^{5} - 1n^{4} + 193n^{3}-\frac {4721}{105}n^{2}-\frac {574}{15}n+24$

### Dispersion

**Fig. 2:**
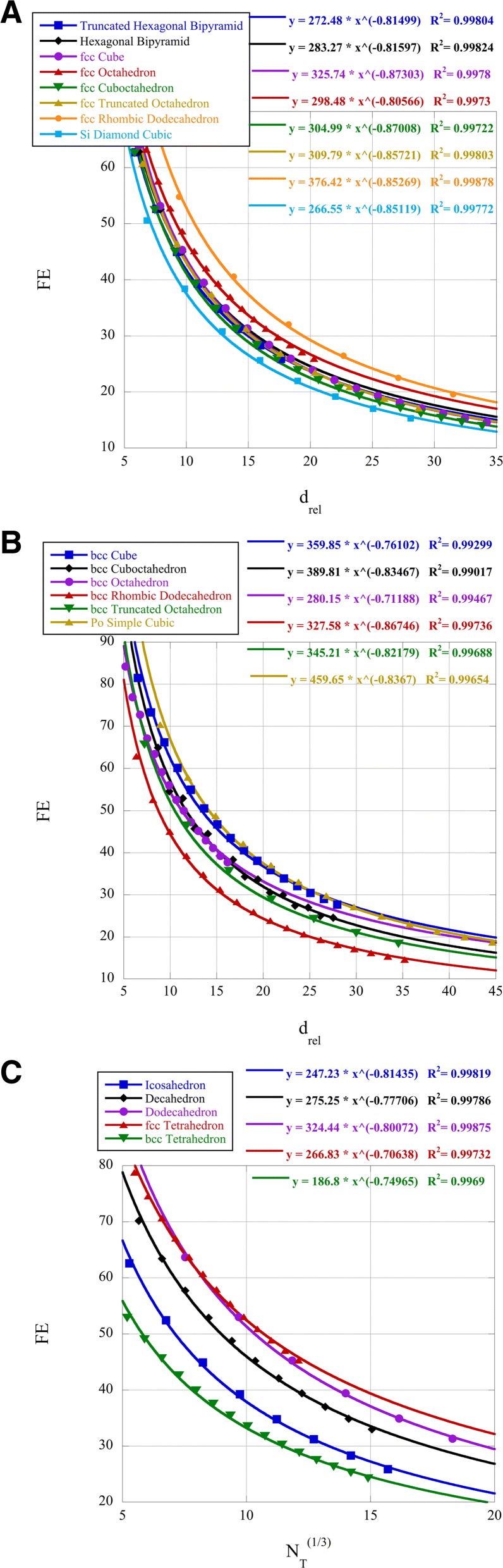
Dispersion FE for the nanoclusters

The percentage of surface atoms (dispersion, FE) of the various clusters is presented in Fig. 2. Platinum nanoclusters are known to have catalytic activity with respect to the oxygen reduction reaction (ORR) which is size and shape dependent [38]. This reference determined that platinum cuboctahedral clusters of 2.2 nm in size had maximal ORR activity. It is also known that for PtNi alloys the (111) surface is preferred for the ORR [39]. We compare the icosahedral, octahedral, decahedral, and cuboctahedral clusters for FE at a *d*_*rel*_=7.5 for platinum at 2.2 nm. The icosahedral, octahedral, and decahedral clusters have surfaces with (111) faces. Using the power laws in Fig. 2, we find for the given *d*_*rel*_ that the FE for icosahedral clusters is 47.9%, for cuboctahedral 52.8%, and for decahedral 57.5% and that octahedral clusters have FE=58.9*%*. Thus, based on shape, the octahedral clusters have both the (111) surface and the highest value of FE for a similar size. Both the power law coefficient and exponent are relevant for the determination of FE for small *d*_*rel*_. The mathematical interpretation of the power law exponent gives the physical significance as the relationship of the ordinate, FE, to the abscissae, *d*_*rel*_, or the relative percent change of FE to the relative percent change of *d*_*rel*_. The power law coefficient is simply the value of FE when *d*_*rel*_=1.

Another research group has synthesized platinum alloy icosahedral clusters and compared the activity with octahedral ones [40]. These nanoclusters were about 13 nm in size or *N*=20,000 for octahedral clusters and *N*=15,000 for icosahedral clusters. This produces a *d*_*rel*_=30 for the octahedral clusters and 25 for the icosahedral ones. Using the relevant power laws, this gives FE=18.0*%* for the octahedral and 19.8% for the icosahedral clusters. There is very little difference in FE for this size of the cluster, but the icosahedral cluster has a significant amount of strain due to twinning, which may shift the d-band center, thus affecting the ORR results [40]. However, given the size-dependent data of [38], it may be suggested that smaller clusters would produce still higher ORR data. Indeed, 4 nm Pt_3_Ni octahedra, when doped with Mo, have produced record-high ORR results [41].

## Conclusions

We have studied 19 types of nanoclusters and some relevant magic formulas for the number of atoms, bonds, coordination numbers, and topological indices. These include the fcc, bcc, hcp, the Platonic solids, diamond cubic, simple cubic, and decahedral clusters. The majority of these results are more detailed than previously determined, and a large number are enumerated for the first time. A grand goal of materials-related research is the correlation of structure with properties. This detailed study of the magical relationships for nanoclusters is a step in that direction. An example is the discussion of the dispersion of surface atoms and its relationship to catalytic activity. It is our intention that these results will aid scientists in their studies of nanocluster structure and the associated properties.

## Abbreviations

bcc: body-centered cubic
cif: crystallographic information file
fcc: face-centered cubic
FE: Fraction Exposed, dispersion
hcp: hexagonal close packed
ORR: Oxidation reduction reaction
rW(G): Reverse Wiener Index
Sz(G): Szeged Index
W(G): Wiener Index
WW(G): Hyper-Wiener Index
